# Resonance Raman spectroscopy of Fe–S proteins and their redox properties

**DOI:** 10.1007/s00775-018-1533-0

**Published:** 2018-01-24

**Authors:** Smilja Todorovic, Miguel Teixeira

**Affiliations:** Instituto de Tecnologia Química e Biológica António Xavier, Universidade Nova de Lisboa, Av da República, 2780-157 Oeiras, Portugal

**Keywords:** Resonance Raman spectroscopy, Iron-sulfur proteins, Reduction potential

## Abstract

Resonance Raman spectra of Fe–S proteins are sensitive to the cluster type, structure and symmetry. Furthermore, bands that originate from bridging and terminal Fe–S vibrations in the 2Fe–2S, 3Fe–4S and 4Fe–4S clusters can be sensitively distinguished in the spectra, as well as the type of non-cysteinyl coordinating ligands, if present. For these reasons, resonance Raman spectroscopy has been playing an exceptionally active role in the studies of Fe–S proteins of diverse structures and functions. We provide here a concise overview of the structural information that can be obtained from resonance Raman spectroscopy on Fe–S clusters, and in parallel, refer to their thermodynamic properties (e.g., reduction potential), which together define the physiological roles of Fe–S proteins. We demonstrate how the knowledge gained over the past several decades on simple clusters nowadays enables studies of complex structures that include Fe–S clusters coupled to other centers and transient processes that involve cluster inter-conversion, biogenesis, disassembly and catalysis.

## Introduction

Resonance Raman (RR) spectroscopy is a powerful tool for identification and characterization of the metal (active) site and elucidation of structure–function relationship in metalloproteins and metalloenzymes, including hemic, iron–sulfur, diiron and copper proteins [1, 2]. When the wavelength of the excitation laser coincides with that of an allowed electronic transition of the protein chromophore, the intensities of certain Raman bands become selectively enhanced by several orders of magnitude. In this manner selectivity also becomes enhanced along with the sensitivity, as only the vibrational modes from the chromophore that gives origin to the electronic spectrum become augmented, while those from other parts of the molecule typically cannot be observed under these conditions. In the case of non-hemic metalloproteins, the enhanced modes mainly contain metal–ligand and intra-ligand stretching and bending vibrations, which include amino acid residues or small inorganic ligands [1, 2].

Over the last couple of decades, RR spectroscopy has proved to be an indispensable tool for identification and characterization of Fe–S clusters in proteins and in particular those that are diamagnetic and therefore EPR silent (e.g., [2Fe–2S]^2+^ and [4Fe–4S]^2+^). RR spectra, obtained upon excitation into S → Fe charge transfer electronic transitions (a ligand-to-metal charge transfer transition, LMCT) are sensitive to Fe–S cluster type, configuration and symmetry and nature of the ligands. The large body of work on synthetic clusters and simple monocluster containing proteins, mainly those involved in electron transfer (ET) (vide infra 3.1) led to a well-established assignment of their vibrational spectra and paved the way to studies of more complex systems [3–10]. More recently, RR has been shown to be capable of providing fine details on transient processes that involve Fe–S proteins [3–10], and has been extended to surface enhanced RR (SERR) to characterize immobilized Fe–S enzymes interacting with physiological substrates [11, 12]. Here, we aim to present an overview of RR studies on structurally and functionally different Fe–S proteins, highlighting the type of information that can be extracted from RR spectra and in parallel, discuss their thermodynamic properties (e.g., reduction potential). After “Introduction”, we will first briefly describe the basics of RR spectroscopy of Fe–S proteins, together with the most commonly used approaches for determination of their redox properties. Then we will focus on the major contributions of RR spectroscopy in the studies of diverse Fe–S proteins, such as those that participate in ET [1, 13–21], DNA repair [11], biogenesis of Fe–S clusters [3, 22–30] and heme cofactors [31], substrate binding and activation, S-donation and catalysis [32–35], and regulation of gene expression [5, 8, 10, 36–39]. We will conclude with several examples of recent RR studies of enzymes carrying complex polychromophoric clusters [7, 40, 41].

### Resonance Raman spectroscopy

**Fig. 1:**
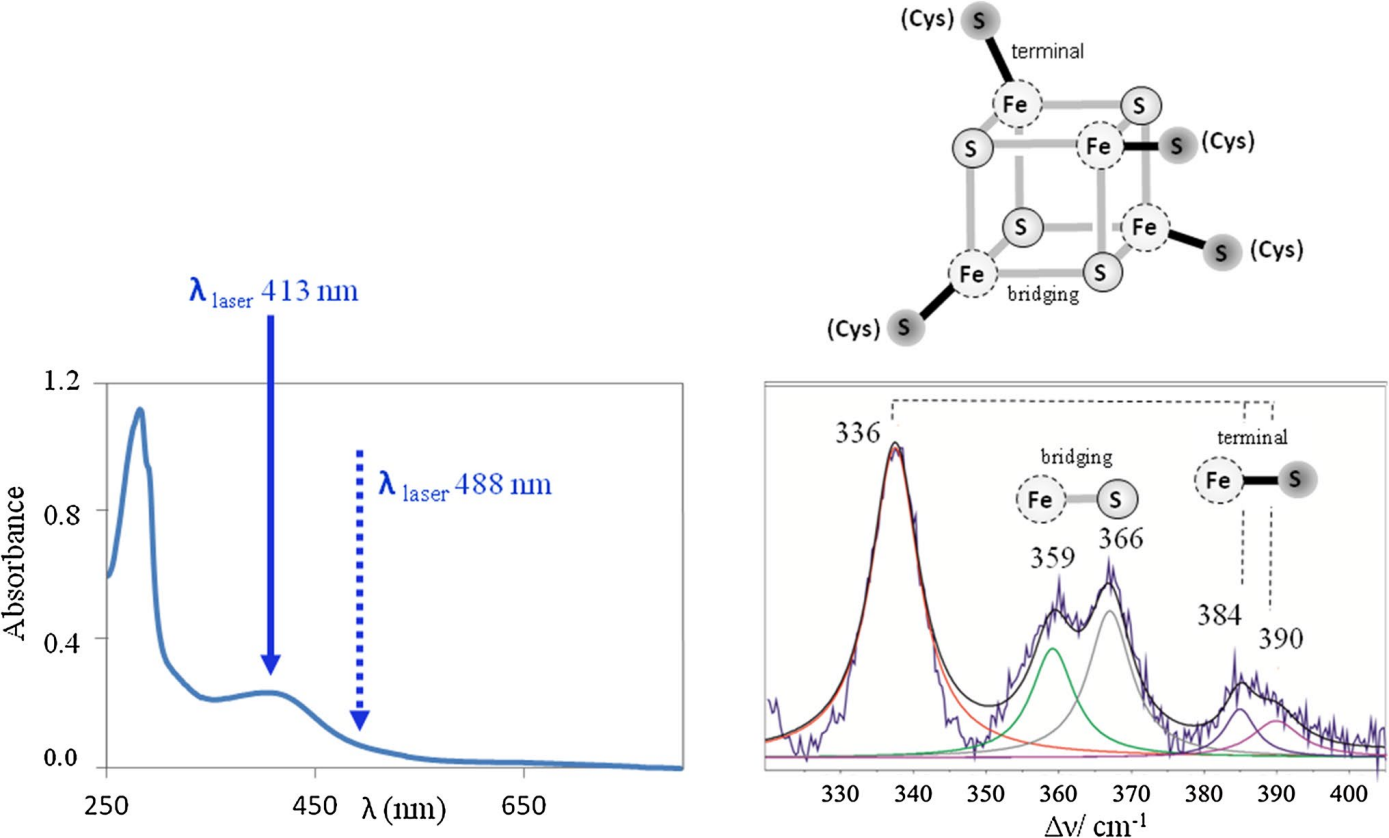
UV–Vis and RR spectra of a [4Fe–4S]^2+^ cluster protein. Left panel, UV–Vis spectra with designated laser excitation wavelengths for resonance and pre-resonance enhancement of the signal. Right panel, experimental and deconvoluted component RR spectrum, with Fe–S bridging and Fe–S(Cys) terminal vibrational modes indicated in the spectrum and in the schematic representation of the cluster

RR spectra of Fe–S cluster containing proteins, obtained with a laser of wavelength that matches the energy of S → Fe charge transfer transitions (Fig. 1) selectively enhances modes involving the metal–ligand stretching coordinates, which can be observed in the low-frequency (200–450 cm^−1^) region [1]. RR spectra of distinct cluster types are well understood due to a thorough pioneer work on isotopically labeled (^54^Fe and ^34^S) proteins and synthetic model compounds, and normal mode analysis, which has been performed mainly by Spiro and co-workers [1, 14–16, 18–20, 35, 42]; the assignments of Fe–S vibrational modes still largely rely on these data. There are several crucial premises that were established in those early experiments. First, different types of clusters have distinct RR fingerprints [1]. As demonstrated by systematic substitutions employing ^34^S in both cluster and terminal thiolate positions, bridging (Fe–S)^b^ and terminal (Fe–S)^t^ vibrational modes (involving inorganic sulfur and cysteinyl sulfur ligands, respectively) can be distinguished in the spectra (Fig. 1) [1, 15, 18–20, 35]. They can therefore be used as a sensitive internal probe for monitoring processes and interactions that involve these specific bonds in a cluster. However, RR spectra of distinct proteins that carry the same type of cluster may show some variations. These variations can be directly correlated with protein-specific (but typically minor) differences in Fe–S bond strengths and Fe–S^t^–C–C dihedral angles, which govern the extent of mixing between cysteinyl S^t^–C–C bending and (Fe–S)^t^ stretching modes and the complexity of the spectra [1, 16]. Second, RR spectra are sensitive to the nature of Fe ligands (besides the most common S ligand from Cys, these may include N provided by His and Arg, O originating from Asp, Ser, Glu or Tyr and exogenous ligands), immediate molecular surrounding (polar vs. hydrophobic) and hydrogen bonding network [1, 13]. Third, a vast majority of the published RR studies concerns analysis of oxidized clusters, since their LMCT transitions have higher intensity (and are consequently more colored). For instance, the broad electronic transition band of ferric 2Fe–2S ferredoxin (Fd), centered around 400 nm, is twice more intense than that of the reduced (Fe^3+^/Fe^2+^) form. Among obvious exceptions are High Potential Iron–sulfur Proteins (HiPIPs), which are RR active in the oxidized and reduced states. Besides, there are also other examples (mainly 2Fe–2S cluster containing proteins) for which RR spectra were reported both for the oxidized and reduced states [42]. Fourth, by a proper choice of excitation wavelength, RR spectroscopy can simultaneously probe different types of clusters in the same protein. For instance, the [3Fe–4S]^1+^ and [4Fe–4S]^2+^ centers of 3Fe–4S/4Fe–4S Fd from *Acidianus ambivalens* (*Aa*Fd) can be simultaneously observed in RR spectra measured with 413 nm laser line, while the spectra measured with 458 and 514 nm selectively enhance the [3Fe–4S]^1+^ center [43].

The possibility to identify the cluster type, its ligands and geometry, paved a way to RR investigations of numerous and diverse Fe–S proteins that incorporate the common 2Fe–2S, 3Fe–4S and 4Fe–4S clusters and unusual heteronuclear complexes that integrate Fe–S clusters. Furthermore, they led to remarkable studies of dynamic processes that involve cluster inter-conversion, biogenesis, disassembly and catalysis; a particularly large body of work on these transient systems comes from Johnson’s group and co-workers [3, 5, 8, 10, 13, 22–28, 30, 32–34, 36–39, 44].

### Redox properties

**Fig. 2:**
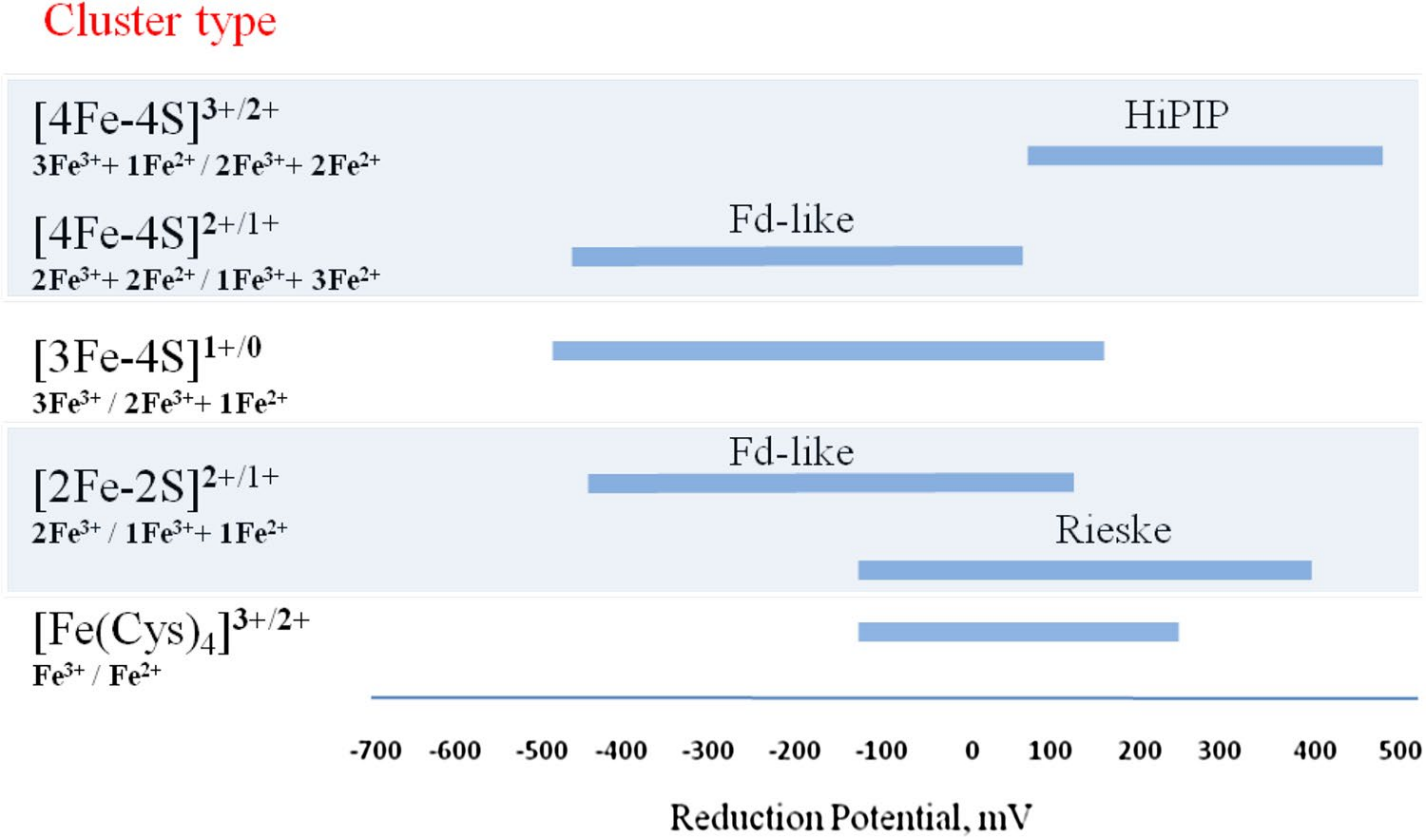
Fe–S cluster redox states and iron formal oxidation states, and reduction potential range for Fe-S containing proteins

Iron–sulfur centers are intrinsically redox active, with the iron ions shuttling between the ferrous (2+) and ferric (3+) oxidation states. Multinuclear clusters have different combinations of ferric and ferrous ions, and are generally described by total cluster oxidation states obtained by adding the formal charges of the inorganic sulfide anions (2−) and of the ferric or ferrous ions (Fig. 2). As the cluster nuclearity increases, the number of possible oxidation states of the cluster increases as well: for example, while for the simplest Fe–S protein, rubredoxin (Rd), only two redox states (ferric and ferrous) are accessible, for cubane clusters up to four states may be reached. However, physiologically, and in contrast with model compounds, in most Fe–S proteins only two redox states are functional, i.e., the interactions between the cluster and the protein backbone are such that the other states are highly thermodynamically unfavoured, transforming the metal centers into simple one-electron transfer agents. Thus, 2Fe–2S centers shuttle between the 2+ (all ferric) and 1+ (ferric/ferrous) stages; a super-reduced form (all ferrous) was electrochemically generated for the special case of the Rieske protein from the *bc*_1_ complex [45, 46]. Trinuclear clusters shuttle between the 1+ (all ferric) and 0 (2 ferric, 1 ferrous ions) oxidation states, and again by electrochemical methods a super-reduced form was obtained in single and dicluster Fds. The transition from the 1+ state to the ‘all ferrous’ 2- state involves three electrons and three protons [47–49]. For the tetranuclear clusters, two types of redox pairs exist: the HiPIP-type, for which the [4Fe–4S]^3+/2+^ redox states are accessible, and the Fd-type proteins, which can stabilize the [4Fe–4S]^2+/1+^ states. For the HiPIPs from *Rodophila globiformis* and *Rhodospirillum salinarum*, a form with EPR characteristics similar to reduced Fd-type clusters, i.e., the super-reduced [4Fe–4S]^1+^ state, was obtain using the strong Ti(III)citrate reductant under basic conditions, at very low redox potentials [50]. The only example of an all-ferrous [4Fe–4S]^0^ center has been reported for the nitrogenase iron–protein [51, 52]. In all cases, the physiological significance, of the super-reduced clusters remains to be established, mainly because of the very low redox potentials at which they are formed (roughly in the − 650 to − 700 mV range).

The reduction potentials of Fe–S proteins span almost the entire physiological range of biologically relevant redox potentials, with a parallel only in heme proteins; this variation illustrates well the fundamental role of the protein environment in fine-tuning the reduction potentials, irrespective of the cluster type (Fig. 2). Two comprehensive reviews on the redox properties of Fe–S proteins are available [53, 54], therefore only a brief overview will be presented here. In Rds and Rd-like domains in more complex proteins, the reduction potentials vary roughly between − 140 and + 250 mV; the lowest value was observed in the Rd-domain of flavorubredoxin from *Escherichia coli* [55] and the highest in the Rd-domain of rubrerythrins [21, 56]. Differences in the reduction potentials of Rd centers have been attributed to variations in hydrogen bonds between the cysteine sulfurs and the amide groups, and to the presence of charged residues close to the center. The larger changes in reduction potentials were obtained by mutating the cysteines to serines, resulting in a 100–200 mV decrease, which was attributed to the high electronegativity of the serinate ligand leading to a higher stabilization of the oxidized form [57, 58]. Dinuclear centers of the Rieske-type proteins (in which one of the iron ions is coordinated by two histidines, instead of the usual cysteines) have reduction potentials between − 150 and + 450 mV. The negative potentials are in general associated with the so-called Rieske Fds involved in dioxygenase reactions, while the positive potentials characterize Rieske proteins of the respiratory/photosynthetic *bc*_1_ and *b*_6_*f* complexes. In the latter, the reduction potentials are strongly pH dependent, and the reduction process occurs with the concomitant uptake of at least one proton, involving the histidine(s) ligand(s). The all-cysteinyl 2Fe–2S clusters have negative potentials, in the range − 100 to ~ − 450 mV. The difference between these and Rieske proteins has been attributed to the substitution of negatively charged cysteines by the neutral histidine imidazoles that stabilize less the oxidized cluster states. Trinuclear clusters cover also a wide range of reduction potentials, from about + 150 to ca − 500 mV, an interval similar to those of the Fd-like [4Fe–4S]^2+/1+^ centers. In contrast, the [4Fe–4S]^3+/2+^ transitions occur at positive potentials, ca. + 50 to + 450 mV, which was rationalized in terms of structural features, the protein fold and immediate cluster surrounding, and in particular the lower number of amide–cysteinyl sulfur hydrogen bonds in HiPIPs as compared with Fd-like proteins.

## Methodologies

### RR spectroscopy of Fe–S clusters: experimental concerns

Raman spectroscopy is a scattering technique which, like infrared (IR) spectroscopy, probes the vibrational levels of the molecule [2]. Vibrational frequencies are sensitive to bond strength and number, geometry and coordination of atoms, and therefore provide information on molecular structures. In Raman spectroscopy the sample is irradiated (i.e., excited) with a highly monochromatic light source from a laser. Only a small fraction of the incident photons undergoes inelastic or Raman scattering, thus emerging from the sample with a different energy. The photons of shifted energy are collected together with the incident laser light, and upon filtering out of the latter, represented in the form of a Raman spectrum. Raman spectroscopy is not quantitative as the band intensity depends on the Raman cross section of the molecule. Moreover, it suffers from low sensitivity, which is due to the low quantum yield of the scattering process (< 10^−9^). This disadvantage can be overcome for molecules that possess chromophoric cofactors through the resonance Raman (RR) effect [2]. In this case, matching of the energy of the incident laser light with an electronic transition of the chromophore increases the quantum yield of the scattering process by several orders of magnitude for the vibrational modes originating from the chromophore. The obtained enhancements of the Raman bands are typically around 10^3^–10^5^, which ensures an increase of the sensitivity and the selectivity of RR spectra that display only the vibrational modes of the cofactor, regardless of the size of the protein matrix. It is noteworthy that the excitation energy does not have to match sharply the electronic transition; the laser wavelength can be as far as 50–100 nm from the absorption maximum of a chromophore, and still provide so-called “pre-resonance” enhancement (Fig. 1). Moreover, if the studied protein possesses multiple chromophores, which have distinct electronic transitions, it is in moderately complex systems often possible to selectively probe individual centers by a careful choice of excitation wavelength. For instance, one can separate contributions from Fe–S cluster and diiron site in rubrerythrins using 568 and 496 nm excitations, respectively [17]; however, RR will not provide quantitative information about different types of centers due to distinct excitation profiles. Further huge enhancement of the signal can be achieved if the protein molecules are found in a close proximity of a nanostructured coinage metal (e.g., on Ag or Au electrodes) or a nanoparticle. In the case when the electronic transition of the molecule matches the laser wavelength (RR) and the energy of surface plasmons of the metal (surface enhanced Raman, SER), these two effects combine to give origin to surface enhanced RR spectroscopy (SERR). In comparison with RR, the sensitivity of SERR increases by another couple of orders of magnitude for the immobilized molecules [2, 59].

Good-quality RR spectra of Fe–S cluster proteins typically require small amounts (microliter volumes) of highly concentrated sample (low millimolar concentrations), a confocal Raman spectrometer equipped with excitation laser line that fulfills the RR condition, e.g., 406, 413, 458, 488 or 514 nm lines from krypton and argon ion laser, and relatively high laser power at a sample (often a couple of milliwatts); note that in setups in which the spectrometer is not coupled to a microscope (which ensures a high power density) these values can be > 100 mW. Relatively high sample concentrations are required due to typically low extinction coefficients of the electronic transitions of Fe–S cofactors; the enhancement factors roughly follow the intensities of the absorption spectrum. The sample is most commonly measured in the frozen state (e.g., 77 K), which ensures narrow bandwidths. A common configuration consists of a N_2_(l)-cooled cryostat that carries a sample droplet, which is mounted on a microscope stage, allowing spectra collection in backscattering geometry. SERR condition is fulfilled for Fe–S proteins attached to Ag surfaces and 413 nm laser; so far there are very few reports on SERR of Fe–S proteins [11, 12].

After polynomial background subtraction, RR spectra are typically treated by component analysis software, which allows for accurate determination of positions, widths and intensities of the individual bands. The region of interest is in the low-frequency (200–450 cm^−1^) range, since the bands found above this interval contain overtones and combination bands; the most intense band typically originates from totally symmetric mode (Table 1). The band positions and relative intensities are characteristic of the particular type and symmetry of the cluster, while the band widths typically reflect homogeneity of the sample. When two distinct clusters are present in the sample, the relative intensities of their RR bands depend on respective Raman cross sections and enhancement factors.

**Table 1:** The common Fe–S cluster types, RR active oxidation states and the most prominent bands, *ν* (cm^−1^). The wavenumber interval in which the predominant RR band is found for each cluster type and the respective symmetry are designated

Cluster type	[Fe(Cys)_4_]^3+/2+^	[2Fe–2S]^2+/1+^	[3Fe–4S]^1+/0^	[4Fe–4S]^3+/2+/1+^	
RR active redox state	**3+**	**2+**	**1+**	**2+**	**3+**
Predominant RR band *ν* (cm^−1^)	314–318 (A_1_)	Rieske: 360 (B_2t_^t^) Fd: 281–291 (B_3u_^t^)	346–348 (A_1_^b^)	HiPIP/Fd: 333–339 (A_1_^b^)	HiPIP: 341–344 (A_1_^b^)

### Reduction potential and its determination

The reduction potential is a key property of the cluster in Fe–S containing proteins, enabling to establish the nature of intra- and inter-molecular ET chains. The reduction potentials of Fe–S proteins have been determined basically by two types of experiments: spectroscopically monitored redox titrations, in the presence of redox mediators that assure the redox equilibrium between the protein and the electrodes, and electrochemical methods, namely cyclic voltammetry (CV).

The electronic transitions of Fe–S proteins in the UV–Vis region are relatively weak and, furthermore, when multiple clusters are present, they result in a broad, featureless spectrum that hinders the assignment of individual clusters. The situation is much worse if the protein under study has other chromophores, namely heme centers, which have molar extinction coefficients in the visible region approximately ten times higher than Fe–S centers. Furthermore, most redox mediators also have reasonably intense visible spectra, and therefore their concentration and type have to be carefully optimized, without compromising the redox equilibrium with the electrodes. Redox titrations are routinely monitored by UV–Vis spectroscopy in the case of the simplest Fe–S proteins like Rds that have characteristic absorption bands in the 380–570 nm range in the ferric state, which are bleached upon reduction, and 2Fe–2S containing proteins. The technique of choice for the determination of the redox properties of more complex Fe–S proteins has been electron paramagnetic resonance (EPR) spectroscopy [55, 60], based on methods originally developed by Dutton [61]. Fe–S proteins have paramagnetic states, which generally display intense and characteristic EPR signals, dependent on the cluster type and oxidation state. The titrations are also performed in the presence of redox mediators [55], but since they do not interfere with the EPR signal, their concentrations may be higher and, which is particularly relevant for membrane-bound proteins (e.g., respiratory complexes) the titrations can be performed using membrane suspensions. Electrochemical methods, namely CV, nowadays most commonly employing carbon-based working electrodes and direct attachment of proteins, are also widely used for the determination of reduction potentials of Fe–S proteins [62–64]. The electrochemical response is often enhanced in the presence of polycations, such as neomycin or polymyxin, when the proteins are negatively charged. Other methods rely on the adsorption of the protein onto Au or Ag metal working electrode surface, either bare or modified employing, e.g., SAMs (self-assembled monolayers), which facilitate protein adsorption via specific interactions and ensure biocompatibility [59]. While electrochemical methods may require small amounts of proteins, they do not allow the assignment of the detected signals to specific clusters in a protein, which is only achieved by EPR spectroscopy.

## RR spectroscopy of Fe–S proteins and enzymes

### Electron transfer

As mentioned above, due to a remarkable chemical versatility of both Fe and S, Fe–S clusters can access various redox states [1, 65]. Consequently, one of the primary roles of Fe–S clusters in proteins is carrying out and mediating biological ET. These functions can be undertaken by FeS_4_, 2Fe–2S, 3Fe–4S and 4Fe–4S clusters in soluble and mobile proteins such as Rds, Fds and HiPIPs, or by clusters incorporated in domains of larger, multi-cofactor containing complexes such as respiratory chain Complexes I, II and III, hydrogenases and photosynthetic (e.g., cytochrome *b*_6_*f*, photosystem I) ET complexes. While the principal role of 2Fe–2S and 3Fe–4S clusters is the transfer of one electron, 4Fe–4S clusters carry out other diverse functions besides ET. A thorough analysis of structures of different Fe–S proteins revealed that folds that integrate low potential [2Fe–2S]^2+/1+^ and [4Fe–4S] ^2+/1+^ clusters represent a vast majority among all Fe–S proteins, while only a very minor number of folds accommodate HiPIPs, Rieske-type and Rd proteins [66].

**Fig. 3:**
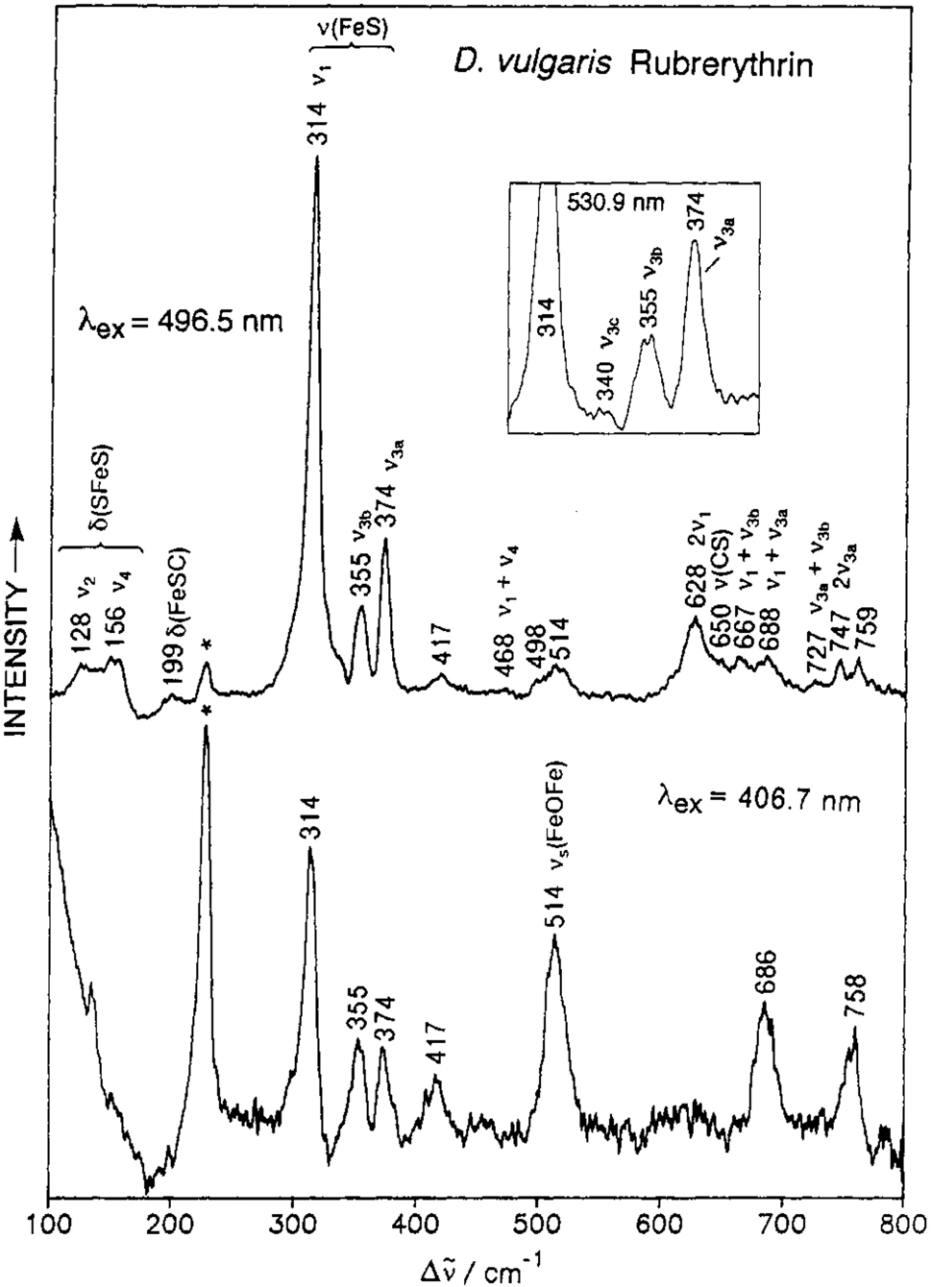
Low-temperature (77 K) RR spectra of as-isolated *Desulfovibrio vulgaris* rubrerythrin obtained with 496 nm (upper trace) and 406 nm (lower trace) excitation wavelengths; the inset shows the ν_3_ Fe–S stretching region excited at 530 nm. The bands in 300–400 cm^−1^ interval represent Rd fingerprint. Reprinted with permission from Dave et al. [17]. Copyright (1994) American Chemical Society

*Rubredoxin*, Fe(Cys)_4_ ([1Fe]^3+/2+^, 3+ state is RR active) is a small ET protein, the simplest among Fe–S proteins. It houses a single Fe ion coordinated by four cysteine sulfur atoms in distorted tetrahedral geometry (D_2d_ symmetry). RR spectra of ferric Rd show a characteristic (not necessarily resolved) four-line pattern in the 310–380 cm^−1^ range (Fig. 3), which originate from Fe–S stretching modes that involve mostly symmetric and asymmetric changes of the four Fe–S(Cys) bond lengths [14]. Overtones and combination bands appear at higher frequencies. The most intense band, found at around 315 cm^−1^, Table 1, is attributed to totally symmetric breathing mode of the FeS_4_ tetrahedron; the bands of significantly lower intensities, centered at ~ 360 cm^−1^, originate from the triply degenerate asymmetric Fe–S stretching [16]. Full understanding of the coupling between Fe–S stretching with S–C–C bending modes in Rd came from normal mode analysis [67].

Rd center is found in more complex proteins, like rubrerythrins, desulforubrerythrins and flavorubredoxins. The spectral contributions of the two chromophores present in rubrerythrins, the Rd and μ-oxo-bridged diiron centers, could be identified in RR spectra measured with 406 and 496 nm excitations. In the case of the latter, RR spectra are dominated by the modes of FeS_4_ tetrahedron (Fig. 3), with the symmetric stretch at 314 cm^−1^ and upshifted low-intensity asymmetric modes. The RR spectrum measured with 406 nm excitation, on the other hand, shows diminished intensities of Rd bands and a pronounced band at 514 cm^−1^, sensitive to ^18^O isotopic substitution, which is characteristic of Fe–O–Fe stretch of oxo-bridged iron (Fig. 3) [17]. RR helped establish the types of cofactors in desulforubrerythrin, which was predicted to have three Fe binding structural domains. Broadened, overlapping bands at 316, 366 and 383 cm^−1^, observed with 413 nm excitation, were indicative of two Fe–(SCys)_4_ clusters. A simultaneously detected mode at 520 cm^−1^, which underwent 17 cm^−1^ downshift upon exchange of ^16^O with ^18^O, was assigned to the μ-oxo-bridged diiron center [21].

**Fig. 4:**
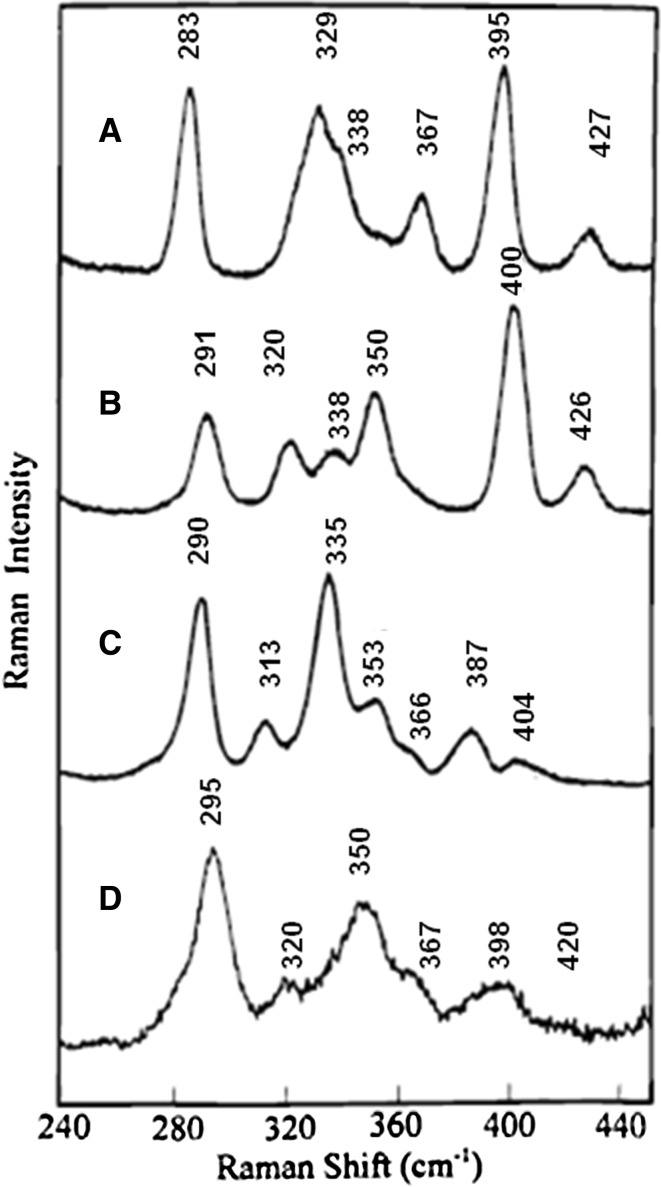
Comparison of the RR spectra of oxidized human ferrochelatase (**d**) with three classes of [2Fe–2S]^2+^ Fds: *S. oleracea* Fd (**a**), *P. putida* Fd (**b**), and *C. pasteurianum* Fd (**c**), recorded at 17–25 K. Reprinted with permission from Crouse et al. [31]. Copyright (1996) American Chemical Society

*Ferredoxins* carry out ET in different biological pathways, including photosynthesis and respiration, employing structurally different Fe–S cofactors. The cluster is most commonly surrounded by hydrophobic residues, but the protein surface typically carries acidic patches that facilitate interaction with physiological redox partners [54]. Fe_2_S_2_(Cys)_4_ clusters (i.e., [2Fe–2S]^2+/1+^; 2+ state is RR active, and less frequently 1+ state) are found in plant-type Fds (e.g., chloroplast Fd I) and mammalian and bacterial proteins, such as adrenodoxin and putidaredoxin. The iron is coordinated by two inorganic sulfur atoms and four cysteine thiolates in distorted tetrahedral geometry (idealized D_h_ or C_2h_ symmetry) [1, 18, 19]. It is noteworthy that the assignments of iron ligand vibrational modes depend on the selected model, which may influence the interpretation of RR spectra; in particular, normal mode calculations based on models with lower symmetry can lead to overestimated Fe–S(t)–C–C dihedral angles and Fe–S bond strengths [68, 69]. RR spectra of Fds are more complex and also more informative than those of Rds, since the vibrational modes that originate from mainly bridging and mainly terminal stretching modes can be distinguished in the spectra [19]. The bridging modes typically have higher energy (315–425 cm^−1^) than the terminal ones (290–360 cm^−1^). In RR spectra of ferric Fds the predominant mode (B_3u_^t^) is found around 290 cm^−1^ (Fig. 4), which is characteristic for all cysteinyl-coordinated clusters that are observed within the 281–291 cm^−1^ interval (Table 1). The presence and intensity of this mode, which should be Raman inactive due to idealized D_2h_ symmetry of the center, could be rationalized in terms of hydrogen bonding in the immediate cluster environment that affects its symmetry. The other intense features of RR spectra of 2Fe–2S clusters are two close modes around 320–340 cm^−1^ (B_1g_^b^ and A_g_^t^) and/or at 390–400 cm^−1^ (A_g_^b^) [19, 42]. RR bands are approx. 10 cm^−1^ upshifted in the clusters with an oxygenic ligand substituting one of the cysteines, which is associated with mass difference between S and O atoms. Reported RR spectra of ferrous 2Fe–2S Fds reveal 15–40 cm^−1^ band shifts to lower frequencies and similar relative intensities as in the spectra of ferric proteins [42].

Fds of bacterial-type house a cubane-like Fe_4_S_4_(Cys)_4_ cluster ([4Fe–4S]^2+/1+^; 2+ state is RR active in Fds and also in HiPIPs, vide infra). Evolutionary, they represent the first ubiquitous ET centers in the majority of anaerobic bacteria [54]. Typically, the most intense RR band of 4Fe–4S Fds is breathing bridging (Fe–S)^b^ mode of the cube at ~ 336 cm^−1^ (A_1_), which in the case of all cysteinyl-coordinated clusters falls into the 333–339 cm^−1^ range (Table 1). The terminal modes are found around 360 cm^−1^ (and 390 cm^−1^). A replacement of conserved Cys by an Asp residue in Fd from *Pyrococcus furiosus* accounts for a subtle 4 cm^−1^ upshift of the A_1_^b^ breathing mode, while a replacement by a Ser residue results in a band upshift characteristic for [4Fe–4S]^2+^ clusters with oxygenic ligands (340–343 cm^−1^ range of A_1_) [13].

**Fig. 5:**
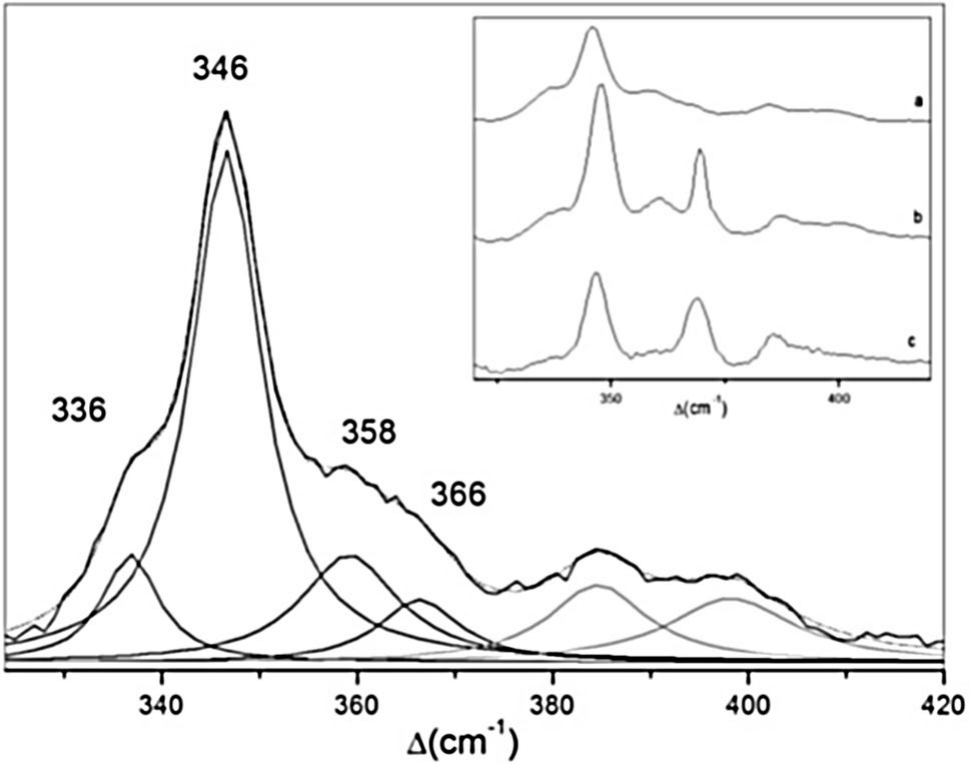
Experimental and band-fitted RR spectra of oxidized 3Fe–4S/4Fe–4S Fd from *Acidianus ambivalens*. Experimental RR spectrum (solid line) was obtained with 413 nm excitation and laser power of 9 mW at 77 K. Overall fitted spectrum: dotted line. Component spectra: (4Fe–4S)^b^ at 336 cm^−1^, (3Fe–4S)^b^ at 346 cm^−1^, (4Fe–4S)^t^ at 358 cm^−1^ and (3Fe–4S)^t^ at 366 cm^−1^; non-assigned bands (grey); inset: comparison of RR spectra measured with 413 (**a**), 514 (**b**) and 458 nm (**c**) excitation. Adapted with permission from Todorovic et al. [43]. Copyright (2006) American Chemical Society

As a result of evolutionary gene modifications, Fds may possess [3Fe–4S] clusters in domains that have lost conserved Cys residues or multiple iron–sulfur centers (e.g., [4Fe–4S]/[3Fe–4S] or [4Fe–4S]/[4Fe–4S]) [54]. Characteristic RR signature of oxidized Fe_3_S_4_(Cys)_3_ cluster ([3Fe–4S]^1+/0^; 1+ state is RR active), present in e.g. Fd II from *Desulfovibrio gigas*, consists of bridging modes that include an intense band at 347 cm^−1^ and weaker ones at 266 and 285 cm^−1^, and terminal modes at 368 and 390 cm^−1^ [20]. The most intense band is the symmetric A_1_^b^ at 346–348 cm^−1^ (Table 1). Distinct RR fingerprints of [4Fe–4S]^2+^ and [3Fe–4S]^1+^ clusters allowed for direct observation of 3Fe–4S intermediate formation upon oxidative degradation of 4Fe–4S cluster in Zn-containing Fd from *Sulfolobus* sp. [70] and independent monitoring of the clusters in Fds that contain both cluster types, such as Fd from the thermoacidophile *A. ambivalens* (*Aa*Fd) [43]. In the latter, differential enhancements of the bands from the two clusters was achieved employing several laser lines (e.g., 413, 458 and 514 nm) (Fig. 5). The most prominent RR band is at 346 cm^−1^ for all three excitation wavelengths, which can be attributed to (3Fe–4S)^b^ mode [44]. The [4Fe–S] cluster was observable only with 413 nm excitation, as judged by the presence of characteristic 336 cm^−1^ band of the (4Fe–4S)^b^ mode that appears as a shoulder in the spectra (Fig. 5). Similarly, in other [4Fe–4S]/[3Fe–4S] cluster containing Fds (e.g., Fds from *Azotobacter vinelandii*, *Thermus thermophilus* and *Sulfolobus* sp.), the most intense is the (3Fe–4S)^b^ band, regardless of the excitation wavelength [20]. The spectral contributions of the two centers in *Aa*Fd were accurately determined by spectral deconvolution, which in addition to more intense bridging modes revealed the presence of terminal modes: (3Fe–4S)^t^ at 366 cm^−1^ and (4Fe–4S)^t^ at 358 cm^−1^ (Fig. 5). The possibility of simultaneous observation of the two clusters by RR spectroscopy allowed for monitoring of their individual thermally induced disassembly at the level of respective bridging and terminal bonds, in a work in which Fd was used as a model for metalloprotein (un)folding study. It was demonstrated that the two clusters disassemble simultaneously, triggering subsequent major structural changes of secondary structural elements of the Fd [43].

*Rieske centers* are Fe_2_S_2_(Cys)_2_(His)_2_ clusters (i.e., [2Fe–2S]^2+/1+^; 2+ state is RR active) that possess a unique ligation motif involving two terminal histidine residues, in addition to two cysteinyl and two bridging sulfur atoms. They are found in *bc*_1_ complex in mitochondria and bacteria and *b*_6_*f* complex of photosynthetic chain in chloroplasts, where they act as primary electron acceptors, and in some soluble bacterial dioxygenases [54]. Due to lower (C_2v_) symmetry of the cluster, RR spectra of these proteins have a higher number of bands in the 200–450 cm^−1^ region than 2Fe–2S Fds. The most intense is the (Fe–S)^t^ stretching mode (B_2t_), which is found around 360 cm^−1^ (Table 1). Other unique features of RR spectra of Rieske centers include pH (i.e., imidazole protonation state) sensitive Fe-imidazole stretching (Fe–N(His)), around 270 cm^−1^ and Fe–N bending modes at lower frequencies. RR spectra of Rieske-type proteins can therefore provide fine details about H bonding network around the cluster, which plays an important role in minimizing the reorganization energy and facilitating ET in these proteins [71]. Different RR spectra and their distinct interpretation were reported for Rieske protein of the toluene-4-monooxygenase complex (T4MOC) from *Pseudomonas mendocina* KR1, in which the most intense RR band was observed at 408 cm^−1^ and assigned to B_2u_^b^ [70]. Extensive kinematic couplings of iron ligand vibrations, both of Fe–S^t^ stretching with δ(Cys) bending and of Fe–S(Cys) stretching with Fe-His vibrations, were observed by measuring N isotope effect of uniformly labeled protein. They were interpreted in terms of rigidity of terminal Fe ligands, imposed by hydrogen bonding and backbone constraints to ensure minimized reorganization energy for ET in T4MOC [70].

*HiPIPs* house a [4Fe–4S]^3+/2+^ cluster; both 2+ and 3+ states are RR active. They are found mainly in photosynthetic anaerobic bacteria, where they act as an electron carrier between the *bc*_1_ complex and the photosynthetic reaction center, but also in aerobic bacteria, where they function as electron donors to terminal oxygen reductases. They are, therefore, to some extent comparable to the better known monohemic cytochrome *c*. RR spectra of reduced HiPIPs are quite similar to those of oxidized, isoelectronic Fds, with the most intense A_1_^b^ band at ~ 336 cm^−1^. The overall complexity of the spectra may be increased by specific protein-induced distortions of the cube [1, 15]. RR spectra of oxidized HiPIPs are also dominated by the totally symmetric bridging vibration of the Fe_4_S_4_ core, which is found at ~ 341–344 cm^−1^ (Table 1) [72]. Other pronounced bands (all terminal) appear at ~ 373, 382–390, 393–403, 410 and 417–415 cm^−1^. The observed upshift of the A_1_^b^ mode (relative to the value for the reduced protein) is rationalized in terms of the overall shortening of Fe–S^b^ bonds. Otherwise, RR spectra of the oxidized HiPIPs from different organisms appear to be remarkably similar and also comparable to those of the respective reduced forms [72].

### DNA repair

There is a growing evidence for the presence of 4Fe–4S clusters in enzymes that take part in nucleic acid processing machinery [73]. These include DNA repair enzymes, such as damage-specific DNA glycosylases: endonuclease III (*End*oIII), MutY and their homologue enzymes found in many organisms including humans, as well as primases, helicases, transcription factors, polymerases and RNA methyltransferases [74]. In the majority of these enzymes the role of the cluster remains to be elucidated [75].

**Fig. 6:**
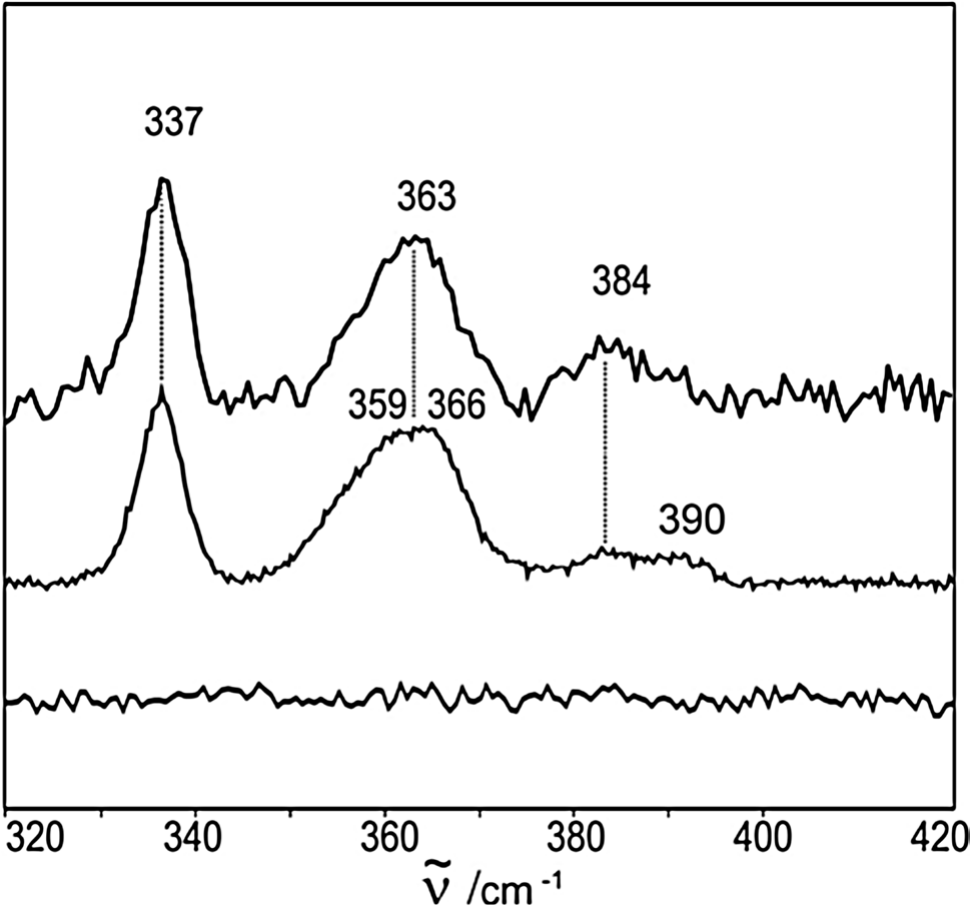
SERR and RR spectra of *End*oIII. SERR spectrum of *End*oIII from *Deinococcus radiodurans* immobilized on Ag electrode modified with mercaptoundecanoic acid-terminated SAM (top trace), upon reduction with sodium dithionite (bottom trace); RR spectrum of *End*oIII in frozen solution (middle trace). Spectra were measured with 413 nm excitation at 77 K using 1.5 and 8 mW in the SERR and RR experiments, respectively. Reproduced from Ref. [11] with permission from The Royal Society of Chemistry

The function and redox properties of 4Fe–4S cluster in *End*oIIIs have been particularly controversial, as the early studies indicated that the cluster could not be chemically oxidized or reduced in solution, suggesting that it had a structural role [76]. RR spectra of *End*oIII from *E. coli* revealed that the cluster is [4Fe–4S]^2+^, with the most prominent bands at 337, 361 and 388 cm^−1^. Subsequent electrochemical experiments indicated that the enzyme becomes redox active upon immobilization on electrodes coated with DNA-terminated SAMs, which led to a speculation that in the presence of DNA the cluster shuttles between 2+ and 3+ states, as in HiPIPs [77]. More recent evidence provided by RR and SERR spectroscopy coupled to electrochemistry, demonstrated that the [4Fe–4S]^2+^ cluster in *End*oIII from *Deinococcus radiodurans* actually shuttles between 1+ and 2+ states, as in Fds, and that its redox activation is not necessarily DNA dependent [11, 12]. Deconvolution of the spectra allowed identification of the bands at 337, 384 and 390 cm^−1^ ((Fe–S)^b^) and at 359 and 366 cm^−1^ ((Fe–S)^t^) in the RR spectra and at 337, 363 and 384 cm^−1^ in SERR spectra (note that in the latter two modes are not resolved in SERR spectra) (Fig. 6). A comparison of the spectra demonstrates that *End*oIII retains its solution structural integrity (characterized by RR) upon immobilization via strong electrostatic interactions with either DNA or COO^−^ modified Ag electrodes (characterized by SERR). Furthermore, reduction of the 4Fe–4S cluster of immobilized *End*oIII resulted in absence of spectroscopic signal, which could be an indication of formation of reduced SERR-silent state but also of protein degradation/desorption. The SERR signal was fully recovered upon re-oxidation, demonstrating that the immobilized *End*oIII was in the +1 state upon reduction. An attempt to oxidize the protein and promote the +3 state, resulted in its irreversible degradation and loss of both electrochemical (CV) and spectroscopic signals. These findings that clearly demonstrate that the cluster shuttles between +1 and +2 states were further supported by structural properties of *End*oIII. Namely, according to the systematic analysis of the known secondary structural elements and folds of proteins that carry [4Fe–4S] clusters (thoroughly examined and discussed in [66]), *End*oIIIs belong to the group of Fd-like low redox potential 4Fe–4S proteins. Taken together, this new spectroscopically based evidence demands for a revision of the mechanistic model of *End*oIII glycosylases derived from electrochemical data.

### Fe–S cluster biosynthesis

**Fig. 7:**
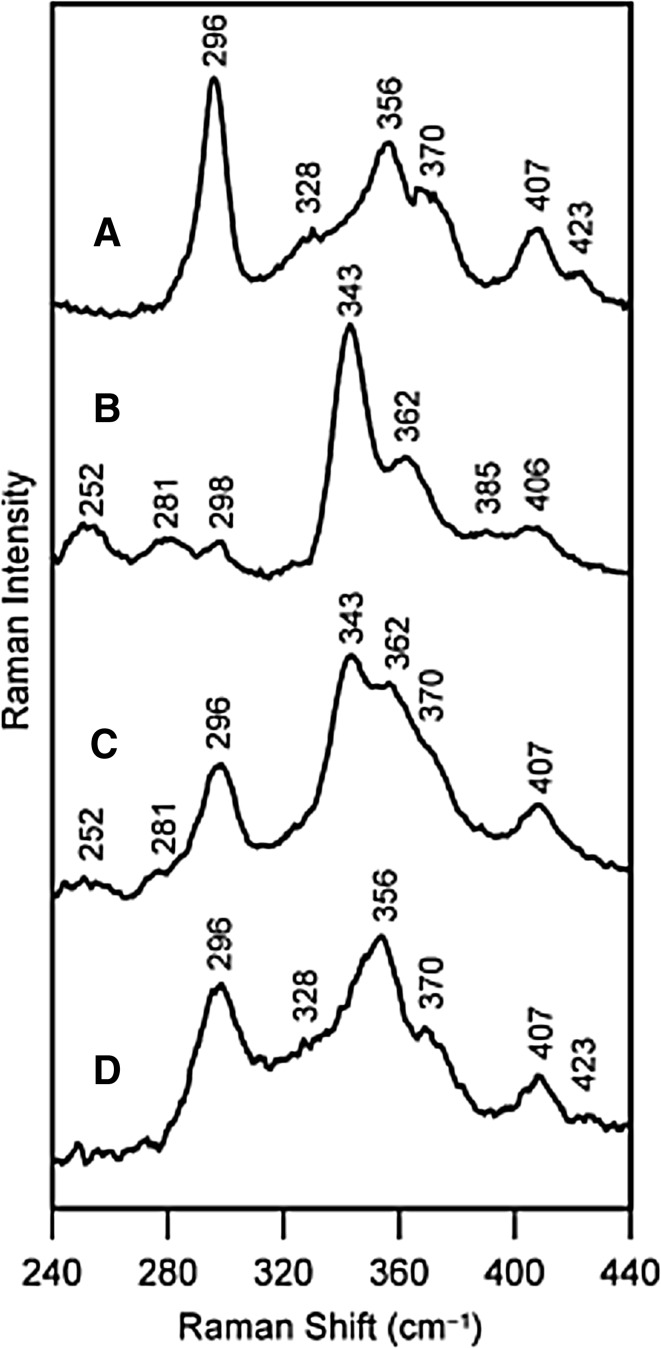
Oxygen-induced [4Fe–4S]-to-[2Fe–2S] cluster conversion on IscU monitored by RR spectroscopy. RR spectra of **a** 2 × [2Fe–2S]^2+^ IscU, **b** [4Fe–4S]^2+^ IscU, **c** [4Fe–4S]^2+^ IscU after exposure to O_2_ for 1 min, and **d** [4Fe–4S]^2+^ IscU after exposure to air for 1 min. Spectra were recorded using 458 nm excitation and 100 mW laser power at 16 K. Reprinted with permission from Chandramouli et al. [26]. Copyright (2007) American Chemical Society

Fe–S cluster biosynthesis has been an active field of research in the last two decades, and RR spectroscopy, in combination with other spectroscopic techniques (mainly UV–Vis, EPR and Mössbauer), helped disentangling several steps of this complex process, which is remarkably conserved in prokaryotic and eukaryotic organisms [3, 65]. Relying on previously established fingerprints of different cluster types and the assignment of their vibrational modes, RR has more recently provided molecular details that are crucial for identification and characterization of the key players in the three types of bacterial Fe–S cluster assembly machinery (NIF, ISC and SUF) [65]. They all show the common cysteine desulfurase-mediated assembly of transient clusters on scaffold proteins and subsequent transfer of pre-formed, solvent exposed clusters to cluster acceptor apo proteins of diverse structures and functions [3, 65]. It is nowadays well established that in all three cases a cysteine desulfurase (IscS, SufS and NifS) provides sulfur for the biosynthesis. However, molecular details on (1) the nature of possible donors of Fe; (2) the dynamics and inter-conversion of transiently formed clusters on scaffold proteins (e.g., IscU, SufU/SufB and NifU; note that SufU is present in *Bacillus* and *Ralstonia* species and SufB in gram-negative bacteria) and their transfer to target proteins; (3) the role of various accessory proteins, including ubiquitous A-type proteins (NifA, NufA, IscA and SufA) in Fe–S cluster biogenesis and maturation, together with (4) the regulation of this process, are still not well established [3, 22–30, 65, 78]. RR studies, particularly those performed by Johnson and co-workers, have contributed to the current understanding of the clusters biogenesis at different levels. First, RR has been employed in investigations of isolated components of the machinery, such as NifU and its truncated/analogous versions IscU and Nfu [23]. The former possesses a permanent redox [2Fe–2S] cluster and a site that serves as a scaffold for NifS-directed assembly of a transient cluster, while IscU can only accommodate the transient cluster. Similarly, the nature and properties of the clusters assembled on chloroplast-specific Nfu2-type protein and its putative physiological cluster acceptors were characterized by RR spectroscopy [6]. The Nfu proteins represent a class of cluster carriers in numerous organisms and possess modular structure which includes a domain analogous to C-terminal domain of NifU. Like bacterial NfuA and human mitochondrial Nfu, Nfu2 from *Arabidopsis thaliana* was shown to be capable of assembling both, [2Fe–2S] and [4Fe–4S] clusters, which it transfers efficiently to glutaredoxin S16 and adenosine 5′-phosphosulfate reductase, respectively. The latter of the two is thought to be physiologically relevant [6]. The recombinant Nfu2 purified under anaerobic conditions contained [2Fe–2S]^2+^, while a cysteine-desulfurase-mediated anaerobic reconstitution of apo Nfu2 resulted in a form that carried [2Fe–2S]^2+^ and [4Fe–4S]^2+^ clusters. Both clusters revealed anomalously high frequencies for all-cysteinyl coordination (i.e., B_3u_^t^ at 295 cm^−1^ and A_g_^t^ at 343 cm^−1^ in [2Fe–2S] and A_1_^b^ at 344 cm^−1^ in [4Fe–4S]), which was attributed to cluster distortions associated with the subunit bridging environment [6]. Second, RR has been used as tool for monitoring of the dynamics of cluster formation and transfer [22, 65]. A typical basic assay for following of the in vitro cluster biosynthesis consists of a mixture of l-cysteine as a donor of S, IscS that catalyzes this process, source of iron (e.g., ferric iron salt or other Fe donor) and IscU or an alternative scaffold, in a controlled reducing environment. The initial insights into formation of transient clusters were obtained from experiments in which the time course of the cluster assembly in IscU was monitored by RR spectroscopy and other methods, which revealed the nature, properties and stability of the formed cluster(s). It was demonstrated that the initial IscU product contained one [2Fe–2S]^2+^ cluster per dimer, then two of them, which were subsequently fully converted into one 4Fe–4S cluster per IscU dimer [22, 23, 65]. The 4Fe–4S formation in IscU, at first described as sluggish, becomes accelerated in the presence of an efficient electron donor, through a reductive coupling of two 2Fe–2S clusters assembled on IscU [26]. RR spectroscopy provided direct evidence for a gradual transformation of 2Fe–2S into 4Fe–4S, and also for the degradation of 4Fe–4S to 2Fe–2S clusters upon exposure to oxygen (Fig. 7). Importantly, the frequencies of RR bands of 2Fe–2S clusters formed in the presence of oxygen coincide with those of the cluster initially formed in IscU, indicating that the clusters are structurally analogous. This capacity of IscU to accommodate either 2Fe–2S or 4Fe–4S cluster was linked to its role in facilitated maturation of distinct Fe–S proteins in response to redox status of the cell and/or oxygen level. Third, since RR spectra are sensitive to the ligand type and conformation, they have provided valuable information about coordination of the newly formed clusters. For instance, the anomalously high frequencies observed in RR spectra of [2Fe–2S]^2+^ cluster formed in IscU (A_g_^t^ and B_3u_^t^ modes at 356 and 296 cm^−1^, respectively) were indicative of non-cysteine ligation. They furthermore coincided with those reported for 2Fe–2S clusters with one serinate ligand (A_g_^t^ within 332–356 cm^−1^ and B_3u_^t^ within 289–302 cm^−1^ range). The totally symmetric breathing mode of the subsequently formed [4Fe–4S]^2+^ center (434 cm^−1^) is also higher than in all-cysteinyl clusters, Table 1, suggesting a non-cysteinyl ligation at one site or unusual H bonding and/or Fe–S–C–C dihedral angles of the cluster [26]. Fourth, RR spectroscopy has helped to establish the possible roles of A-type proteins in the cluster formation. It was first suggested that IscA can play a role of an alternative scaffold for assembly of [2Fe–2S]^2+^ cluster when IscU was absent from the reaction mixture [78, 79], and subsequently demonstrated that its primary functions include Fe–S shuttling [80] and Fe-donation [28, 81]. Also, in NIF-specific Fe–S cluster biogenesis, IscA (i.e., ^Nif^IscA) was capable of binding one iron atom per homodimer, in a cluster with mixed Cys/Asp coordination, as indicated by RR bands at 298, 338 and 397 cm^−1^ [27, 28]. The Fe^3+^–bound IscA, was shown to be a competent iron source for in vitro NifS-mediated 2Fe–2S cluster assembly on the N-terminal domain of NifU. Furthermore the ^Nif^IscA could be rapidly and reversibly converted from one [2Fe–2S]^2+^ to one [4Fe–4S]^2+^ containing form (per homodimer). The former is formed through O_2_-induced cleavage of the [4Fe–4S]^2+^ cluster, and the latter via two-electron reductive coupling of two [2Fe–2S]^2+^ clusters. Taken together, it was proposed that A-type proteins can play a role of specific iron donors for cluster assembly on U-type scaffolds and for repair of [3Fe–4S]^+^ clusters, and furthermore that they can function as carriers of 2Fe–2S, rather than 4Fe–4S clusters, assembled on U-type proteins to acceptor proteins [27, 28].

### Substrate activation and catalysis

RR has been a crucial experimental tool in the investigations of Fe–S proteins in which the cluster, with typically non-all-cysteinyl coordination, represents a site for substrate binding, such as in radical-S-adenosyl-l-methionine (SAM) enzymes [34] and aconitases [35]. The latter enzymes catalyze the isomerization of citrate to iso-citrate in an active site that houses a [4Fe–4S]^2+^ cluster and directly binds the substrate; both inactive (i.e., [3Fe–4S]^1+^ containing) and the activated forms of the cluster have been characterized by RR and their Fe–S modes assigned [34]. Members of the large family of radical-SAM enzymes catalyze diverse radical reactions in a variety of biosynthetic processes via reductive cleavage of SAM. They typically possess three cysteine residues coordinating three iron atoms of a 4Fe–4S cluster at the active site of the enzyme. The remaining ligand of the fourth, so-called unique Fe, at which substrate binding and activation occur, is in the absence of SAM not known. RR spectroscopy significantly contributed to the current understanding of the physiological state of the [4Fe–4S]^2+/1+^ cluster in the active site and of the nature of the second Fe–S cluster of unknown function, which is present in some of these proteins [32–34]. Namely, due to the incomplete coordination, the [4Fe–4S]^2+^ clusters in proteins from the radical-SAM family are particularly prone to degradation, which led to conflicting data in the literature about the physiologically relevant type/state of the cluster. RR spectroscopy provided crucial evidence about [4Fe–4S] cluster sensitivity, demonstrating its prompt conversion to a quasi-stable [2Fe–2S]^2+^ form under aerobic conditions, which sometimes proceeds via formation of a [3Fe–4S]^1+^ cluster, explaining the presence of [2Fe–2S]^2+^ or [3Fe–4S]^1+^ forms in aerobically purified proteins [32].

RR has been employed, in combination with EPR and Mössbauer spectroscopies, in investigations of the two Fe–S clusters in BioB, the enzyme responsible for converting dethiobiotin to biotin by inserting a sulfur atom between the two carbons of the substrate [32]. It was shown that BioB carries one [2Fe–2S]^2+^ cluster with partial non-cysteinyl ligation (the most prominent bands at 301, 331 and 349 cm^−1^) and one [4Fe–4S]^2+/1+^ cluster, which binds SAM at a unique Fe site (characteristic bands at 338 and 364 cm^−1^). The latter undergoes O_2_-induced degradation via formation of a distinct [2Fe–2S]^2+^ intermediate, with broadened bands at 292, 336 and 398 cm^−1^. RR evidence supported direct interaction of SAM with the unique iron of the [4Fe–4S]^2+^ cluster and indicated that the [2Fe–2S]^2+^ center can act as an immediate S-donor during the turnover cluster transformations [32]. Similarly, RR helped characterize the properties of the Fe–S clusters in MOCS1 that catalyzes the conversion of a guanosine derivative to precursor Z during molybdenum cofactor biosynthesis. It  also helped to validate the controversial work with in vitro reconstituted recombinant enzymes [34], as  the  anaerobically purified MOCS1 differed from the enzyme obtained by in vitro reconstitution of the clusters under anaerobic conditions and from that purified under aerobic conditions. The current  view  is  that  the two [4Fe–4S]^2+^ cluster containing MOCS1 is the catalytically competent form, although it could not be experimentally detected in the intact form due to a rapid degradation in the presence of oxygen to semi-stable [2Fe–2S]^2+^ and [3Fe–4S]^0^ intermediates [34].

### Regulation of gene expression

Fe–S clusters are present in transcriptional and translational regulators of gene expression, in which upon environmental stimuli, they undergo transformation (e.g., cluster assembly, conversion or redox reaction) that triggers respective cellular response mechanisms. In combination with other spectroscopic techniques, RR has revealed the details about the cluster formation, type and coordination along these processes. The studied systems include proteins that are stress-responsive transcriptional regulators, and/or participate in iron metabolism, such as BolA proteins and homologues [8, 39]. Among the latter is Fra2, which plays a key role in regulating the iron homeostasis in yeasts. RR spectroscopy revealed molecular details about the complex formation between Fra2 and cytosolic monothiol glutaredoxins (Grx3/4) via bridging though a [2Fe–2S]^2+^ cluster. The spectra of the Grx3–Fra2 complex indicated a presence of mixed cysteinyl- and histidyl-ligated [2Fe–2S]^2+^ cluster, based on the appearance of two low-frequency modes at 275 and 300 cm^−1^, characteristic of partial histidyl ligation [36, 38]. This type of coordination is nevertheless not exclusive in the complexes formed between BolA (and homologues) with Grx, as all-cysteinyl and Rieske-type [2Fe–2S] clusters could also be identified from RR spectra [8]. RR has, furthermore also revealed a notable similarity between [2Fe–2S]-bridged complexes in yeast and human systems involving human BolA2 and Glrx5 [37].

**Fig. 8:**
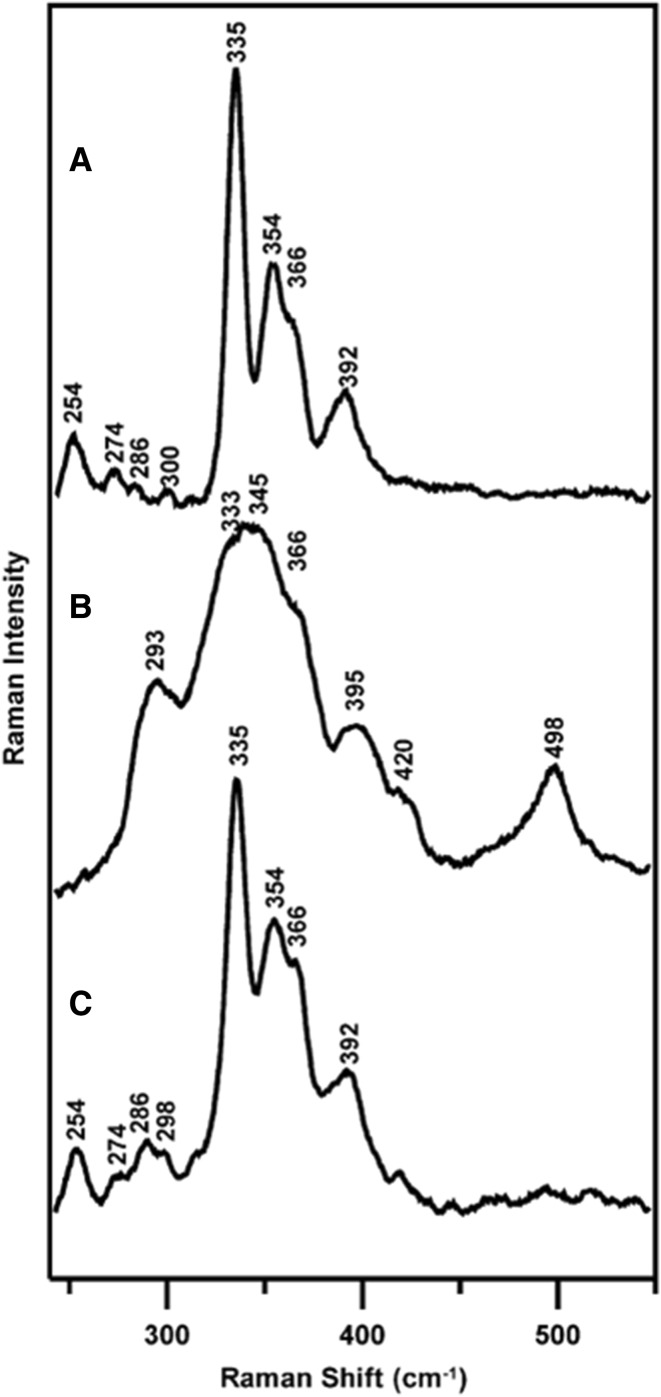
RR studies of the [4Fe–4S]^2+^ and [2Fe–2S]^2+^ inter-conversion in FNR. **a** Reconstituted [4Fe–4S]^2+^–FNR in the presence of GSH. **b** [2Fe–2S]^2+^–FNR obtained by exposing the sample (**a**) to air for 20 min and **c** after incubation of the sample (**b**) with DTT and ferrous ammonium sulfate under anaerobic conditions for 20 min. The spectra are recorded at 21 K with 458 nm laser excitation of 140 mW power. Reprinted with permission from PNAS from Zhang et al. [10]

RR spectroscopy has provided a remarkable evidence for inter-conversion between [4Fe–4S]^2+^ and [2Fe–2S]^2+^ clusters in fumarate and nitrate reduction (FNR) regulatory proteins, which are bacterial O_2_-sensing transcription factors that control the switch between aerobic and anaerobic metabolism [10]. As a response to increased oxygen levels, the [4Fe–4S]^2+^ cluster in these proteins undergoes a rapid conversion to a [2Fe–2S]^2+^ cluster, triggering a dimer-to-monomer transition and loss of site-specific DNA binding. RR spectra of anaerobically reconstituted FNR reveal all cysteine-ligated [4Fe–4S]^2+^ cluster (bands at 335, 354, 366 and 392 cm^−1^) that can be assigned under effective *D*_2*d*_ symmetry, similar to those of 4Fe–4S Fd (Fig. 8). After exposure to air, the cluster is converted into an atypical [2Fe–2S]^2+^ center, which displays low-intensity RR bands at 293, 345, and 395 cm^−1^ that were attributed to cysteine persulfide-ligated [2Fe–2S]^2+^. This conversion of [4Fe–4S]^2+^ to cysteine persulfide-ligated [2Fe–2S]^2+^ cluster in FNR is reversible under anaerobic conditions in the presence of DTT and excess of ferrous iron (Fig. 8). The formation of cysteine persulfide-ligated [2Fe–2S]^2+^ was correlated to O_2_-induced S^2−^ to S^0^ oxidation and the molecular mechanism of O_2_ sensing by FNR, suggesting unique pathways for the assembly and/or repair of biological [4Fe–4S] clusters [10].

Together with optical and Mössbauer spectroscopies, RR has been used to address the controversial views on the type of the physiological cluster in the NO-sensing module of transcription factor NsrR from the Rrf2 family, which controls expression of genes in response to NO in a wide range of bacteria. It was demonstrated that initially observed 2Fe–2S cluster in NsrR was actually an artifact, and that the protein houses a [4Fe–4S]^2+^ cluster, with (Fe–S)^t^ at ∼ 363 and 389 cm^−1^ and (Fe–S)^b^ stretching modes at 343 and 389 cm^−1^, which were assigned by analogy with simple Fd under idealized *T*_*d*_ or *D*_2*d*_ symmetry [5]. The high frequency of the symmetric (Fe–S)^b^, found at 343 cm^−1^, was rationalized in terms of oxygenic ligation in the cluster, with a highly conserved Glu being envisaged as a possible candidate for this ligand [5].

### Fe–S clusters in complex multiple-cofactor centers

**Fig. 9:**
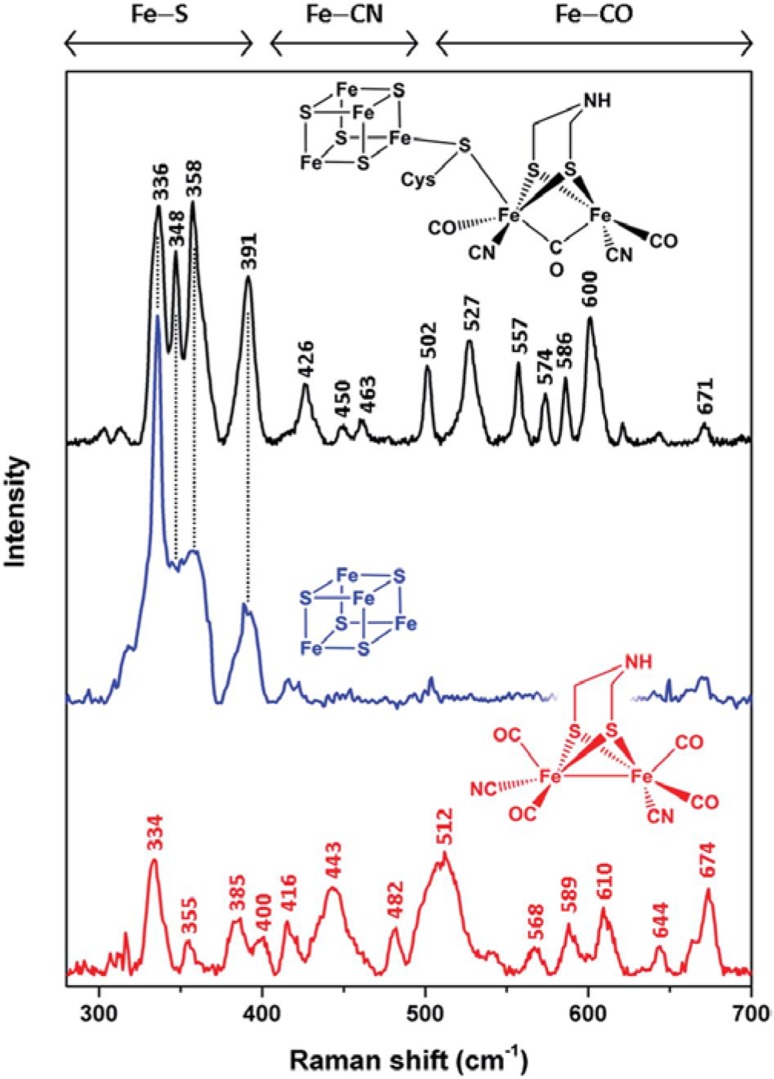
Low-temperature RR spectra of [FeFe] hydrogenase HydA1 and model compounds. The reduced synthetic Fe–Fe–aza-dithiolate complex (red, 514 nm excitation), thionine-oxidized apo-HydA1 (blue, 458 nm), and in vitro-matured holo-HydA1 aza-dithiolate complex (black, 488 nm excitation). Spectral regions reflecting normal modes with major contributions from Fe–S, Fe–CN, and Fe–CO coordinates are indicated. Reprinted from Katz et al. [7]—published by The Royal Society of Chemistry

Due to potential biotechnological applications of hydrogenases for hydrogen production as a clean fuel, intensive research efforts have been made in order to understand the catalytic mechanism of these enzymes, which catalyze reversible cleavage of hydrogen. RR has recently revealed structural insights into catalytic intermediate species in several of these enzymes [7, 82, 83]. Depending on the type and the source, hydrogenases can carry a variable number of different Fe–S clusters that are either essential for the ET and/or together with the binuclear center, constitute the active site. NiFe hydrogenases typically possess three Fe–S centers which conduct electrons from the active site to the physiological redox partner. In combination with theoretical approaches and FTIR spectroscopy, RR provided molecular details on the heterobimetallic active site and the Fe–S clusters in regulatory NiFe hydrogenase (RH) in oxidized and H_2_-reduced states. It has been used to probe the [4Fe–4S]^2+^ clusters, which revealed bands below 400 cm^−1^ that diminish upon H_2_-incubation, indicating reduction to the RR-silent [4Fe–4S]^1+^ state, and to simultaneously monitor the active site (i.e., Fe–CN and Fe–CO stretching modes) that show bands at higher (400–600 cm^−1^) frequencies [82]. Similarly, RR spectra of membrane-bound hydrogenase, from which the contributions of the Fe–S clusters were excluded by H_2_-reduction, revealed modes originating from the heterobimetallic active site that are sensitive to its structure [83]. Furthermore, in combination with FTIR spectroscopy, RR has provided new insights into the nature of catalytic intermediates of a FeFe hydrogenase, which houses a Fd-like [4Fe–4S]^2+^ cluster that is covalently bound via a cysteinyl thiolate link to one of the Fe atoms in the catalytic site. In these enzymes the cluster acts as an electron entry site [7]. RR allowed for simultaneous observation of the [4Fe–4S]^2+^ cluster, displaying 336, 348 and 358 cm^−1^ bands (Fig. 9) and Fe–CN and Fe–CO stretching modes of the active center at higher frequencies, in ‘as-isolated’, H_2_-reduced, thionine-oxidized and CO-bound states of the enzyme. Furthermore, RR was capable of identifying a transient catalytic intermediate, in which the active site is in the mixed valence Fe^+^Fe^2+^ state and the cluster is reduced, [4Fe–4S]^1+^. It was assigned to a deprotonated H cluster intermediate, which is formed first during the biological hydrogen production and had never been experimentally observed before, allowing for establishment of a more complete picture of the catalytic cycle of FeFe hydrogenases [7].

Other proteins that house complex cofactors include sulfite reductases and nitrogenases, among others. The former carry a siroheme, in which the 4Fe–4S cluster is covalently bridged to the heme iron by a cysteinyl S atom [40]. Low frequency of RR spectra obtained with excitation in the siroheme Soret or Q bands are dominated by heme modes, while the 4Fe–4S cluster can be selectively enhanced with 488 or 458 nm excitation. RR spectra are comparable with those of other 4Fe–4S proteins; however, the A_1_^b^ breathing mode was found at 342 cm^−1^, which is significantly higher than that observed in the other analogous clusters (Table 1). Additional ^34^S-sensitive features observed at 352 and 393 cm^−1^ in the spectra of sulfite erductase obtained with Soret-band excitation were putatively attributed to a bridging thiolate ligand [40]. The information that can be extracted from RR spectra on the unique cluster in nitrogenases is still relatively limited, due to inherently weak signals. In fact the first RR spectrum of a nitrogenase from *Azotobacter vinelandii* that houses the ‘FeMo-cofactor’ in the active site (i.e., [Mo–7Fe–9S-Ci], where Ci is carbide), has been reported only recently, employing high laser power and exceptionally long accumulation times. The spectra obtained with 488 nm excitation reveal A_1_^b^ mode at 338 cm^−1^, together with two additional bands 356 and 382 cm^−1^ in nitrogenase [41].

## Outlook

The continuous progress in experimental methodologies that include more efficient protein expression systems and purification methods, alongside with more sensitive and faster spectroscopic techniques, allow us nowadays to identify and characterize Fe–S clusters in exceptionally complex, unstable and transient systems and processes. Due to these advances, we have been able to encounter previously undetected Fe–S clusters in known proteins, to propose new roles for the clusters and to improve our understanding of physiologically relevant and unusually complex cofactors that integrate Fe–S clusters. Being sensitive to the type, ligands and configuration of a cluster, RR spectroscopy has been playing an important part in these discoveries. Some of the recent achievements include the evidence for the presence of a [2Fe–2S]^2+^ cluster in a kinase/phosphatase Asp1 that regulates cell morphogenesis in yeasts [9], characterization of the [4Fe–4S]^2+^ cluster in HydF, a protein involved in the maturation of organometallic H cluster of Fe–Fe hydrogenase [4] and elucidation of the missing pieces (i.e., the transient catalytic intermediates) of the catalytic cycle puzzle in hydrogenases [7, 82], which provide the key information about biological hydrogen activation. We believe that RR spectroscopy has a bright future in illuminating the structure and function of Fe–S clusters that are still to come to our lab benches.
