# Correction to: Resonance Raman spectroscopy of Fe–S proteins and their redox properties

**DOI:** 10.1007/s00775-018-1577-1

**Published:** 2018-06-13

**Authors:** Smilja Todorovic, Miguel Teixeira

**Affiliations:** Instituto de Tecnologia Química e Biológica António Xavier, Universidade Nova de Lisboa, Av da República, 2780-157 Oeiras, Portugal

## Correction to: JBIC Journal of Biological Inorganic Chemistry 10.1007/s00775-018-1533-0

The article “Resonance Raman spectroscopy of Fe–S proteins and their redox properties”, written by Smilja Todorovic, Miguel Teixeira was originally published electronically on the publisher’s internet portal (currently SpringerLink) without open access.

The copyright of the article changed on June, 8 to © The Author(s) 2018 and the article is forthwith distributed under the terms of the Creative Commons Attribution 4.0 International License (http://creativecommons.org/licenses/by/4.0/), which permits use, duplication, adaptation, distribution and reproduction in any medium or format, as long as you give appropriate credit to the original author(s) and the source, provide a link to the Creative Commons license and indicate if changes were made.

The original article has been corrected.

Moreover, the original version of this article unfortunately contained an error in Fig. 1. The correct Fig. 1 is given below.

**Fig. 1:**
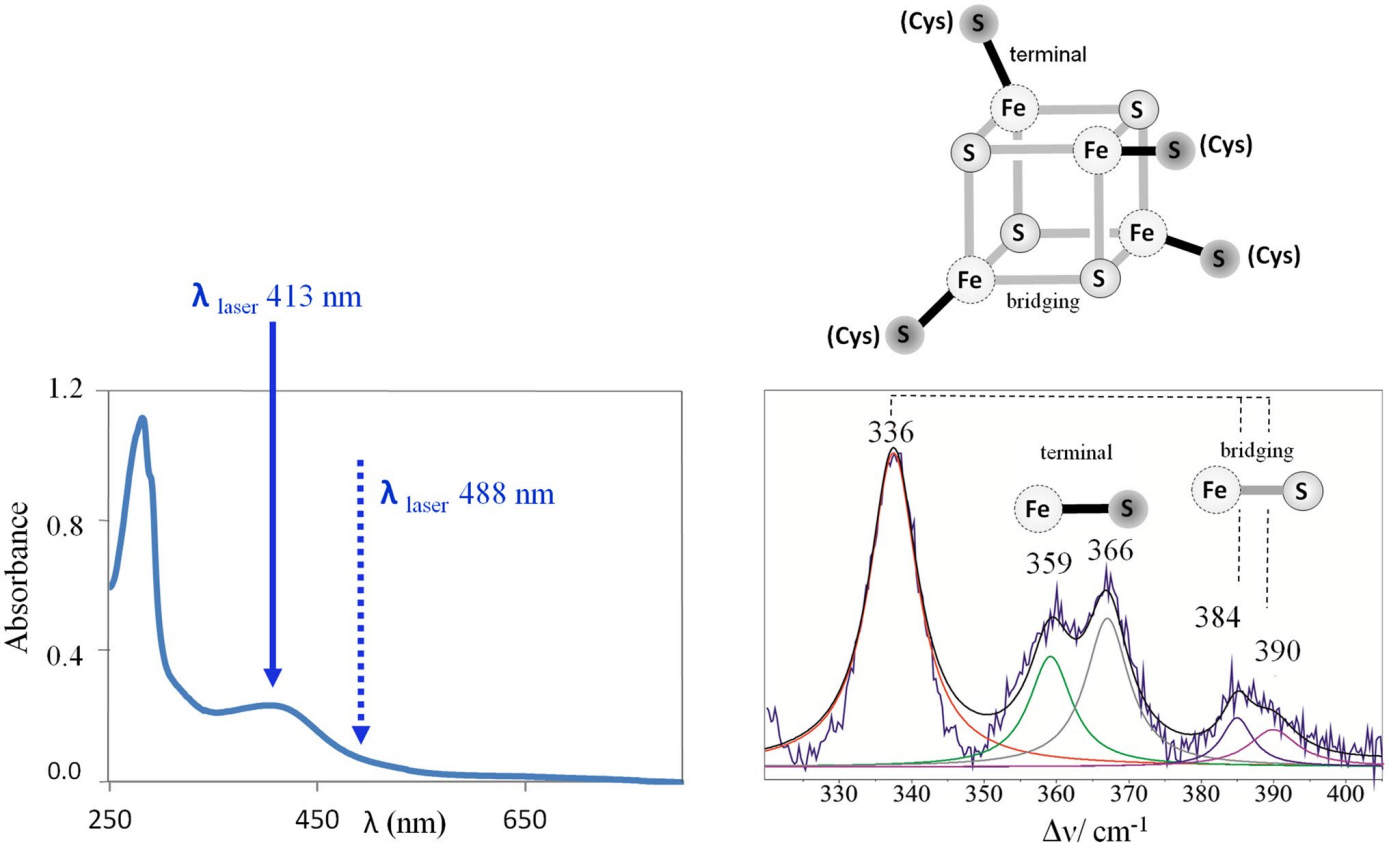
UV–Vis and RR spectra of a [4Fe–4S]^2+^ cluster protein. Left panel, UV–Vis spectra with designated laser excitation wavelengths for resonance and pre-resonance enhancement of the signal. Right panel, experimental and deconvoluted component RR spectrum, with Fe–S bridging and Fe–S(Cys) terminal vibrational modes indicated in the spectrum and in the schematic representation of the cluster

